# Growth and Characterization of Ultrathin Vanadium Oxide Films on HOPG

**DOI:** 10.3390/nano12183134

**Published:** 2022-09-09

**Authors:** Yue Sun, Koen Schouteden, María Recaman Payo, Jean-Pierre Locquet, Jin Won Seo

**Affiliations:** 1 Department of Materials Engineering, KU Leuven, Kasteelpark Arenberg 44, B-3001 Leuven, Belgium; 2 Department of Physics and Astronomy, KU Leuven, Celestijnenlaan 200D, B-3001 Leuven, Belgium

**Keywords:** thin films, 2D, vanadium oxide, HOPG, molecular beam epitaxy, atomic force microscopy, scanning tunneling microscopy, X-ray photoelectron spectroscopy

## Abstract

Integration of graphene into various electronic devices requires an ultrathin oxide layer on top of graphene. However, direct thin film growth of oxide on graphene is not evident because of the low surface energy of graphene promoting three-dimensional island growth. In this study, we demonstrate the growth of ultrathin vanadium oxide films on a highly oriented pyrolytic graphite (HOPG) surface, which mimics the graphene surface, using (oxygen-assisted) molecular beam epitaxy, followed by a post-annealing. The structural properties, surface morphology, and chemical composition of the films have been systematically investigated by in situ reflection high-energy electron diffraction during the growth and by ex situ techniques, such as atomic force microscopy, scanning tunneling microscopy, transmission electron microscopy, and X-ray photoelectron spectroscopy (XPS). Crystalline monolayer vanadium oxide can be achieved on HOPG by systematically tuning the deposition time of V atoms and by subsequent annealing at 450 °C in controlled atmospheres. Increasing the partial pressure of O_2_ during the deposition seems to decrease the mobility of V atoms on the graphitic surface of HOPG and promote the formation of a two-dimensional (2D) vanadium oxide. The obtained oxide layers are found to be polycrystalline with an average grain size of 15 nm and to have a mixed-valence state with mainly V^5+^ and V^4+^. Moreover, XPS valence band measurements indicate that the vanadium oxide is insulating. These results demonstrate that a 2D insulating vanadium oxide can be grown directly on HOPG and suggest vanadium oxide as a promising candidate for graphene/oxide heterostructures.

## 1. Introduction

Graphene is a two-dimensional (2D) material with extraordinary electronic, optical, and mechanical properties and has been explored for application in various fields such as field-effect transistors (FET), photodetectors, and stretchable electronics [1,2,3,4]. In particular, the fabrication of stacked heterostructures of high-quality 2D oxide layers and graphene layers is required to improve the performance of graphene-based electronic devices [5,6]. Two-dimensional oxide thin films with a thickness of only one or two atomic layers are also interesting from a fundamental point of view because they may exhibit novel physical and chemical properties that differ from the corresponding bulk counterparts [7,8]. However, the direct growth of oxide films on graphene is challenging and has scarcely been reported because the low surface energy of graphene strongly promotes the three-dimensional (3D) island growth of oxides [9]. Generally, additional treatments are required to achieve a flat oxide film on graphene. For instance, Wang et al. applied a Ti dressing layer prior to MgO deposition on top of HOPG and graphene to obtain an atomically smooth MgO overlayer [10]. Jandhyala et al. found that ozone molecules are physisorbed on the surface of graphene and can act as nucleation sites for high-κ dielectric deposition (such as Al_2_O_3_) [11]. Thus far, only monolayer yttria (Y_2_O_3_) thin films with a hexagonal lattice have been obtained on graphene by reactive vapor deposition of yttrium in an O_2_ atmosphere [12]. To the best of our best knowledge, other 2D metal oxide thin films successfully grown on graphene have not yet been reported.

Compared to oxides, the growth of a metal on graphene or graphene-like surfaces has extensively been studied, e.g., aiming for improvement and/or manipulation of the electronic and magnetic properties of graphene through metal atom adsorption but also as dressing layers [13]. Based on density functional theory (DFT), Liu et al. calculated the ratio of the adsorption energy to the bulk cohesive energy (E*_a_*/E*_c_*) of metal V on graphene and predicted that V would favor a 3D growth mode on top of graphene [13]. Experimental deposition of V metal on graphene or graphene-like surface has not been reported thus far.

Vanadium (V) is known for its various stable oxidation states (V^2+^, V^3+^, V^4+^, and V^5+^), and the corresponding oxide films possess a range of superior properties which are promising, e.g., for applications in surface oxidation catalysts, gas sensors, supercapacitors, lithium-ion batteries, and nanoelectronics [14,15,16,17,18,19]. Until now, there are only a few reports about thick VO_2_ films (~40 nm thick) grown on graphene on Cu [20] or sapphire substrates [21] for use in flexible thermochromic windows. Ultrathin vanadium oxide (VO*_x_*) films on a graphene-like surface have not been reported.

We use highly oriented pyrolytic graphite (HOPG) substrates which may provide an ideal alternative graphene model substrate for VO_x_ growth studies. Firstly, HOPG consists of (stacked) high-quality defect-free graphene layers. Secondly, due to the weak interlayer coupling, the top layer of the HOPG (that is contaminated due to exposure to ambient air) can easily be peeled off, e.g., using an adhesive tape, hence providing a clean and atomically flat graphitic HOPG surface ideally suited for high-quality material depositions [10,22]. Compared to graphene grown on a metal substrate (e.g., on copper) using chemical vapor deposition (CVD), HOPG allows us to study the direct interaction of the oxide film and the graphitic substrate, without taking, for instance, the presence of polymer surface residues (e.g., for transferred CVD grown graphene), metal particles or wrinkles into account.

In this work, we propose a method to produce a monolayer vanadium oxide film on HOPG by depositing V metal by means of molecular beam epitaxy (MBE) and by applying a subsequent post-annealing in a controlled atmosphere. MBE is particularly favorable for the growth of high-purity films, compared to other techniques such as atomic layer deposition (ALD) [23] and CVD [24], owing to its precise control of the growth condition and RHEED capability for in situ monitoring. Our growth study confirms the island growth of V when metal V is deposited on the surface of HOPG (001), whereas the deposition of V in the presence of molecular oxygen (O_2_) promotes the formation of 2D VO*_x_*. These samples are annealed in argon, and characterization techniques involving atomic force microscopy (AFM), scanning tunneling microscopy (STM), and X-ray photoelectron spectroscopy (XPS) are applied for the in-depth characterization of the resulting films to reveal the growth mechanism. To the best of our knowledge, this is the first report about 2D VO*_x_* on a graphene-like surface, which is of great interest for both fundamental study and technological applications.

## 2. Experimental Section

### 2.1. Thin Film Deposition

HOPG (0001) substrates (10 mm × 10 mm, ZYB grade) were freshly cleaved using an adhesive tape to obtain a clean surface and were subsequently inserted into the oxygen-assisted MBE chamber, which operates at a base pressure near 10^−9^ mbar. The substrate was degassed at 150 °C for 5 min before film growth to remove adsorbed contaminants present on the surface and was kept at 125 °C during the film growth. An electron gun was used to evaporate metallic vanadium at a deposition rate of 0.001 nm/s (calibrated with a quartz crystal microbalance (QCM, INFICON IC/5 Thin Film Deposition Controller, INFICON, East Syracuse, NY, USA). In this way, we have grown metallic V films on HOPG via deposition in ultra-high vacuum (UHV) conditions, whereas VO*_x_* films are grown directly by deposition of V in the presence of an O_2_ partial pressure of 1 × 10^−6^ mbar.

### 2.2. Post-Annealing Treatment

After deposition, the as-grown samples were annealed in a horizontal furnace (Carbolite-Gero, Verder Scientific Benelux, Aartselaar, Belgium) with a quartz tube diameter of 2.7 cm under continuous argon (Ar) gas flow at a rate of 0.2 L/min and with residual oxygen background of the order of 2 mbar in the furnace tube [25]. The films were heated from room temperature to 450 °C at a ramping rate of 10 °C/min. The annealing temperature was maintained for 30 min, after which the films were naturally cooled down to room temperature inside the furnace to prevent exposure to ambient air at elevated temperatures. During the entire procedure, the Ar flow rate of 0.2 L/min was maintained inside the furnace. This post-annealing recipe is based on the previous study of Van Bilzen et al. [25], in which they successfully crystallize amorphous VO*_x_* films on Si substrate.

### 2.3. Characterization

During the film deposition, in situ reflection high-energy electron diffraction (RHEED) was used to monitor the film growth process. After the samples were taken out from the MBE chamber, a series of structural, morphological, and chemical characterizations were conducted. AFM (ParkXE-100AFM, Park Systems Corporation, Suwon, South Korea) and STM (NaioSTM; Nanosurf AG, Liestal, Switzerland) were performed in ambient conditions at room temperature. The AFM images were collected in the tapping mode using Si probes. The STM images were recorded in the constant-current mode by using mechanically cut Pt/Ir tips. Image processing and analysis were performed using the open-source software Gwyddion (version 2.52, Nečas and Klapetek et al., Brno, Czech Republic) [26] and WSxM (version 5.0, Horcas and Fernández et al., Madrid, Spain) [27]. The root mean square (RMS) roughness of the sample surface was calculated via the statistical quantity module in Gwyddion [26]. Plane-view transmission electron microscopy (TEM, JEOL ARM200F, Tokyo, Japan) was performed on a small piece of sample exfoliated from the sample surface. XPS was performed in a FlexMod SPECS (SPECS Surface Nano Analysis GmbH, Berlin, Germany) system equipped with a monochromatic 100 W Al source (*E* = 1486.7 eV). The base pressure of the SPECS system is in the 10^−10^ mbar range. XPS spectra were recorded with a step of 0.05 eV and a pass energy of 20 eV. The XPS data were analyzed with the CasaXPS software [28], and a Shirley function was used to subtract the background radiation. Spectra are energy-calibrated relative to the O1s peak (530.0 eV) [29]. In addition, in order to limit the surface contamination to its minimum, all measurements were conducted as soon as possible, and the samples were stored in a vacuum box (in the range of 10^−2^ mbar) between each measurement. Investigation of the role of the substrate temperature (including room temperature) during the film growth, the O_2_ partial pressure, as well as the post-growth annealing temperature, is the scope of our ongoing research.

## 3. Results and Discussions

First, we study the growth behavior of V on HOPG by depositing V as a function of growth time (30 s, 300 s, and 450 s). An additional sample was produced by depositing V under O_2_ partial pressure of 1 × 10^−6^ mbar to study the effect of O_2_ during the growth. Deposition details of those as-grown films are summarized in Table 1. Throughout the manuscript, we will use the subscript ‘_1_’ in the sample names to refer to the as-grown films, whereas the subscript ‘_2_’ refers to the Ar annealed films which will be discussed later.

### 3.1. RHEED Analysis

**Figure 1:**
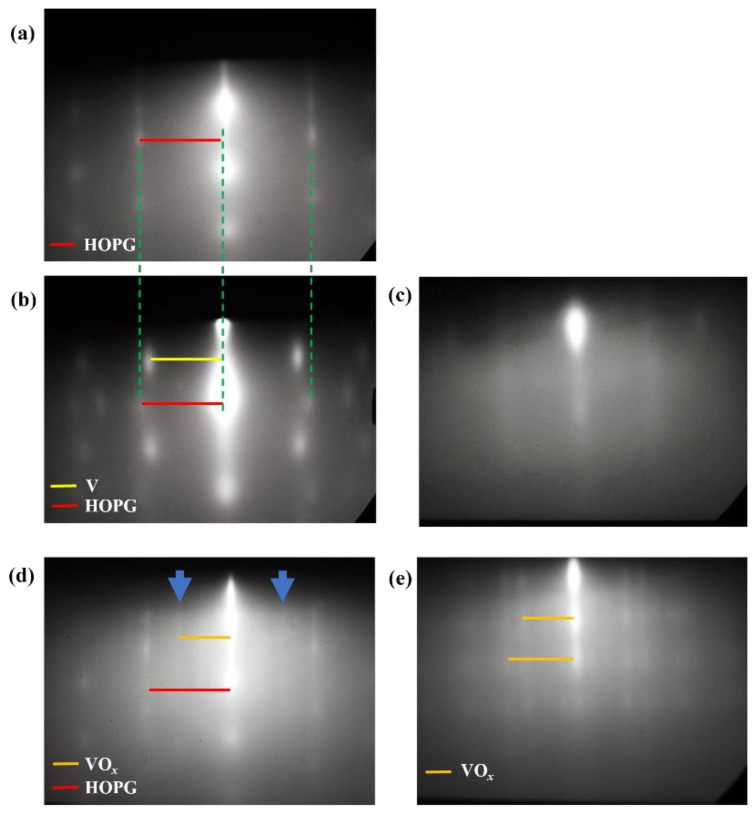
RHEED pattern of (**a**) clean HOPG (after degassing); (**b**) sample c_1_, 0.45 nm V deposition on HOPG without O_2_; (**c**) sample d_1_, 0.45 nm V deposition on HOPG with O_2_; (**d**) annealed sample c_2_; and (**e**) annealed sample d_2_. The yellow, orange and red solid lines indicate the RHEED streak spacings of V, VO*_x_* and HOPG, respectively. The green dotted lines indicate that the RHEED spacings of HOPG are the same before and after V deposition. The blue arrows mark the weak streak patterns from VO*_x_*.

To unveil the initial growth of V or VO*_x_* on HOPG substrate, RHEED was applied for in situ monitoring of the depositions as well as ex situ on annealed samples. Figure 1 presents representative RHEED images at the various involved growth stages. As Figure 1a demonstrates, the RHEED pattern of the pristine HOPG reveals streaks indicating a clean and flat substrate surface after degassing. The distance of the streaks coincides with the spacing of HOPG (100). After deposition of 0.45 nm V on HOPG (sample c_1_), a spot-like pattern from crystalline V appeared (Figure 1b) in addition to the weak streak pattern from the HOPG substrate, revealing that the metal V follows an island growth mode and does not fully cover the substrate. In contrast, the RHEED image of sample d_1_ in Figure 1c shows a diffuse background with weak broad streaks, implying that the film possesses mostly a disordered structure, most probably due to the oxidation of V during deposition.

To further investigate the as-grown samples, a series of ex situ characterization measurements such as XPS, TEM, and AFM were performed. Note that the V sample can easily be oxidized [30,31] after being taken out from the MBE chamber and exposed to air. XPS analysis of the as-grown sample a_1_ indicates a mixed oxidation state of V^1+/2+^, V^4+^, and V^5+^, as shown in Supplementary Figure S1 (top) and Table S1. We assume that the surface of the metal V film is prone to get oxidized to V^4+^ and V^5+^, while the inner part of the metal V film near the HOPG interface is less oxidized and yields the V^1+/2+^-contribution in the XPS spectrum [31]. XPS analysis of depth profiling of the as-grown sample is not performed in our experiment because the low coverage of our VO*_x_* films and the preferential sputtering of oxygen can lead to the creation of lower oxidation states [30,31] which make the depth profiling less meaningful. We also find that, although the RHEED image (Figure 1b) indicates that the metal V is crystallized in the as-grown sample c_1_, its ex situ TEM results (Figure S2) indicate that the metal V film has been oxidized upon exposure to air and formed vanadium oxide with an amorphous structure. Interestingly, in the RHEED image of annealed sample c_2_ (Figure 1d), (weak) streak patterns from crystalline vanadium oxide and HOPG are observed. RHEED image of annealed sample d_2_ (Figure 1e) shows a spot-like pattern from crystalline vanadium oxide indicating that it has an island surface morphology. The diffuse background in Figure 1d,e can be ascribed (at least partially) to the presence of surface contaminants related to ambient exposure. The crystallization and surface morphology of vanadium oxide after post-annealing will be further discussed later combined with AFM and STM analysis.

### 3.2. Surface Morphology

**Figure 2:**
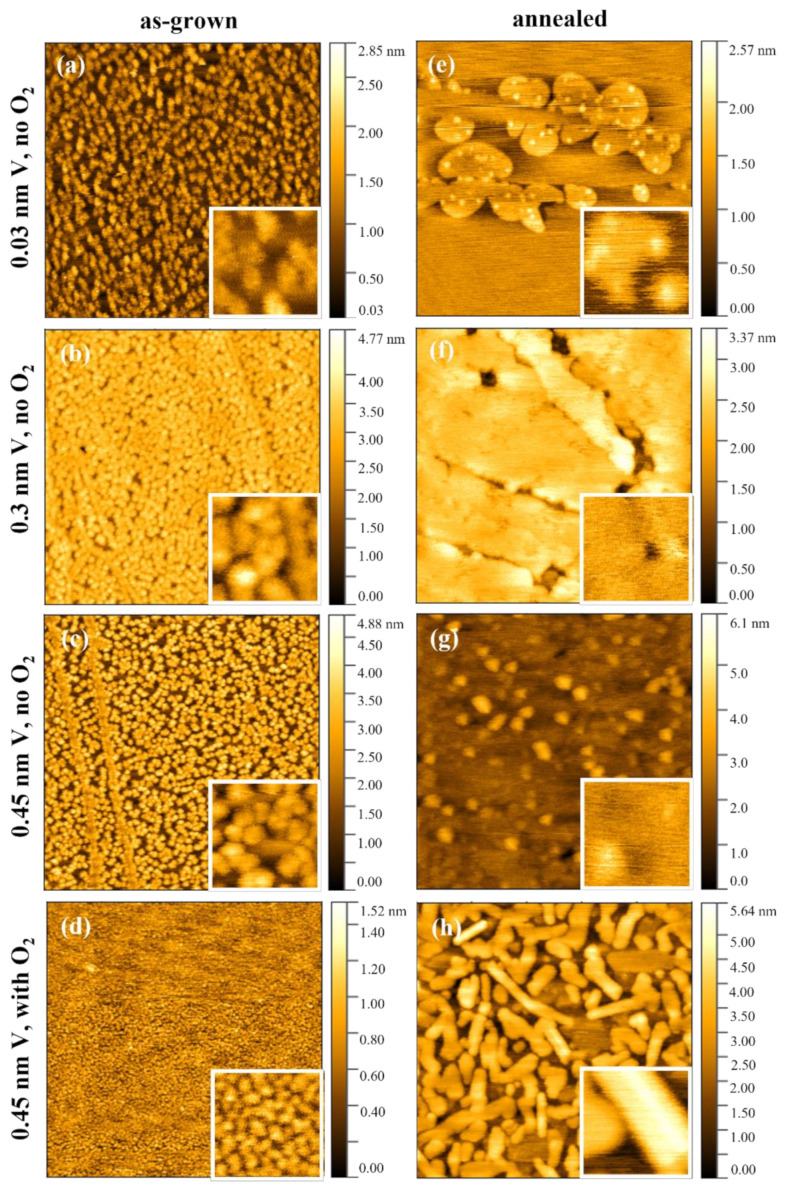
AFM images with size 1 × 1 µm^2^: (**a**–**d**) as-grown samples a_1_–d_1_, and (**e**–**h**) corresponding annealed samples a_2_–d_2_. The RMS roughness of the samples is provided in Table S2. Insets show AFM scans at higher magnification (100 × 100 nm^2^).

As shown in Figure 2a–d, AFM images of those as-grown samples illustrate islands distributed uniformly on top of the terrace of HOPG, which is consistent with the DFT prediction by Liu et al. [13] and our RHEED pattern observation. Additionally, V preferentially forms clusters at the step edges of HOPG which can be observed in Figure 2c. This trend of step-edge decoration on HOPG has widely been reported for various metals due to the higher binding energy or lower mobility of metal atoms near the step edge of HOPG [32]. According to Figure 2a–c, we can conclude that the height, nucleation density, and coverage of the 3D islands increase as a function of deposition time. The accumulation of V during deposition leads to the coalescence of the islands and consequently a higher surface roughness.

Remarkably, by adding a small amount of O_2_ (1 × 10^−6^ mbar) during deposition the roughness can be reduced significantly: the as-grown sample d_1_ (Figure 2d) exhibits a roughness of 0.15 (±0.1) nm, which is much lower than all other three as-grown V/HOPG samples (Table S2). Comparing the inset images of Figure 2d with Figure 2c, it can be concluded that the islands are flatter and smaller, while the nucleation density and coverage of the flattened islands increase significantly. Hence, we assume that O_2_ promotes the formation of 2D VO*_x_* species on top of HOPG by forming chemical bonding with V, increasing nucleation sites, and suppressing the clustering of V atoms to form islands.

To render the film crystalline, all samples were post-annealed at 450 °C in a horizontal tube furnace as described in the Experimental Section, in a condition identified as a protective atmosphere for VO*_x_* [25]. Remarkably, the surface morphology of the samples changes significantly after post-annealing as displayed in Figure 2e–h. With a sub-monolayer coverage of vanadium oxide, annealed sample a_2_ (Figure 2e) shows a morphology with 2D islands accumulating at the step edge of HOPG but barely covering the terrace of HOPG. Interestingly, small bright clusters are present on top of the 2D oxide islands, indicating that V atoms rather prefer nucleating on V island surfaces than on HOPG terraces. Increasing the thickness of the vanadium layer leads to an increase in the lateral 2D island size and hence to coalescence into an approximately full 2D wetting layer covering the HOPG substrate, as presented in the AFM image of the annealed sample b_2_ (Figure 2f). After completion of the first monolayer, the following VO*_x_*-material seems to start a 3D island growth on top of the base layer in thicker films according to Figure 2g (sample c_2_) and Figure 2h (sample d_2_). Therefore, the formation of a full monolayer marks the transition from 2D to 3D oxide growth. Based on these results, we can conclude that the growth of monolayer vanadium oxide on HOPG is evident by depositing V and post-annealing. 

This formation of a monolayer followed by oxidation and annealing of V islands has also been reported on Au (111) [33]. The nanometre-sized islands observed in samples a_2_, c_2_, and d_2_ may result from oxidation at the initial position of the V islands in the as-grown films during annealing. The island morphology of sample d_2_ agrees well with its spot-like RHEED pattern (Figure 1e). The observation that the islands in sample d_2_ are in general wider and flatter than in sample c_2_ is noteworthy. One possible explanation for this morphology difference is that the metallic V layers grown on vanadium oxide are flatter than directly on the HOPG substrate due to a stronger interaction and chemical bonding with the oxide surface which also influences the atomic rearrangement within crystallites and coalescence of crystallites during the oxidation procedure at elevated temperature [34]. This will be further discussed together with the following STM measurements. 

**Figure 3:**
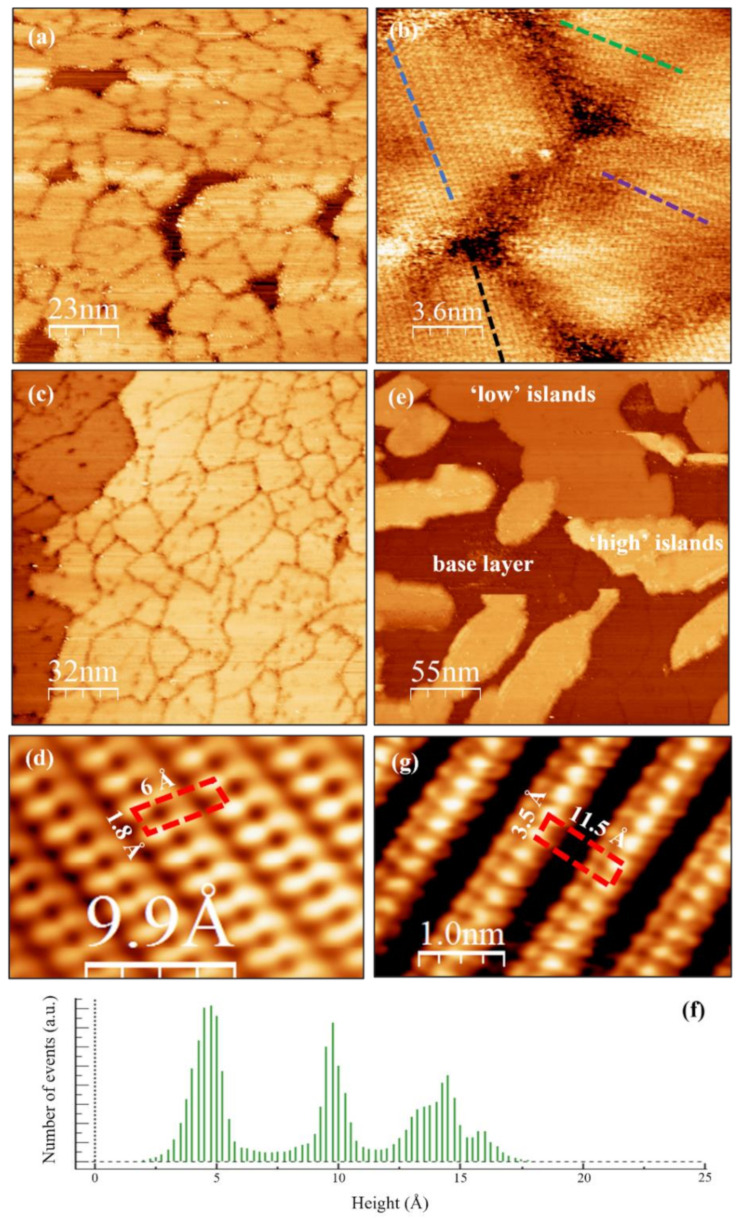
STM characterization of the VO*_x_* films after post-annealing. (**a**) Large-scale and (**b**) small-scale topographies of the annealed sample b_2_. Dotted lines indicate the orientation of various domains. (**c**) Large-scale topography and (**d**) atomic-resolution STM image of the annealed sample c_2_. (**e**) Large-scale topography image of the annealed sample d_2_ and (**f**) the derived height histogram. (**g**) Atomic-resolution STM image of the ‘high’ islands in (**e**). Atomic-resolution images (**d**,**g**) are optimized by applying Fourier-transform-filtering. Scanning bias voltage and current are (**a**,**b**) 0.7 V, 0.6 nA; (**c**) 0.8 V, 0.6 nA; (**d**) 0.8 V, 1.0 nA; (**e**) 0.9 V, 0.6 nA; (**g**) 0.7 V, 0.7 nA, respectively.

To gain more insight into the surface morphology and structure of the ultrathin film, we performed STM experiments on the annealed films. Note that the STM image of sample a_2_ is not shown here because mainly HOPG substrate is observed from its surface, and the small VO*_x_* islands are hardly detected. These islands are also found to be mobile during STM scanning. A large-scale STM image of the annealed sample b_2_ is presented in Figure 3a and shows a flat and almost fully covered oxide layer, in very good agreement with its morphology observed via AFM (Figure 2f). The thickness of the monolayer is around 4.5 Å according to the STM height profile taken across the base layer and the uncovered substrate area (not shown). A higher magnification STM topography image (Figure 3b) reveals that the oxide layer is in fact polycrystalline, consisting of rotational domains with a size in the range of 10 ~ 30 nm. The density of domains of the annealed film is comparable to that of the as-grown sample (inset image in Figure 2b). Figure 3c shows an STM image of the annealed sample c_2_ and reveals a fully covered base layer. The two samples b_2_ and c_2_ exhibit a similar atomic structure with a 1.8 Å × 6 Å oblique unit cell (Figure 3d) which is in agreement with the V_6_O_13_-like structure reported by Guimond et al. [33]. Moreover, this STM observation of small nanosized grains with crystalline vanadium oxide in various orientations is consistent with the weak streak patterns in the RHEED image (Figure 1d) of sample b_2_. 

As illustrated in Figure 3e, the annealed sample d_2_ has a complex morphology with a base layer plus some ‘low’ and ‘high’ islands on top. The height histogram in Figure 3f indicates that those islands occur with heights that are a multiple of about 4.5 Å with respect to the monolayer (the monolayer corresponds to the zero-height value in Figure 3f). Interestingly, atomic-resolution STM images show that the lowest islands atop the base layer exhibit a V_6_O_13_-like structure similar to the base layer itself. In contrast, the higher islands possess a V_2_O_5_ (001)-type structure as illustrated in Figure 3g and exhibit a 3.5 Å × 11.5 Å rectangular unit cell. This type of ‘V_6_O_13_-like base layer + V_2_O_5_ (001) islands’ morphology was also observed on Au (111) surfaces by Guimond et al. when they increased the coverage to 1.56 ML V on Au (111) [33]. It is important to notice that bulk V_2_O_5_ possesses a layered structure, and the neighboring layers are held together by van der Waals force. The thickness of one V_2_O_5_ (001) layer (~4.37 Å) [35] matches well with the measured step height (~4.5 Å) from Figure 3f.

### 3.3. XPS Analysis

**Figure 4:**
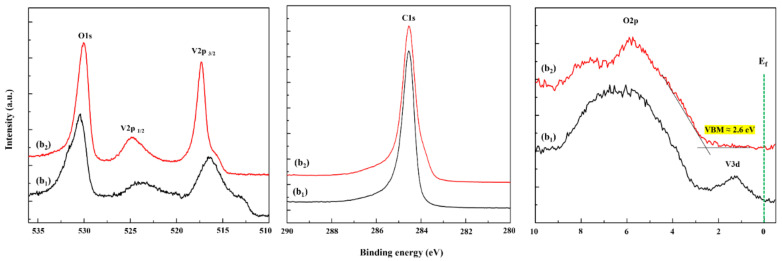
XPS core-level (**left**) O1s-V2p and (**middle**) C1s spectra, and (**right**) valence band spectra of sample b_1_ (as-grown) and b_2_ (annealed).

XPS was further employed to investigate the surface chemical composition of the as-grown and annealed vanadium oxide films. Although the binding energy (BE) of the C1s is widely used for energy calibration, it has been reported that the O1s signal is a better energy reference than the C1s for the V2p BE of vanadium oxide [29,31,36]. Therefore, O1s is used for the BE calibration of the obtained XPS spectra. Core-level O1s and V2p spectra of the samples b_1_ and b_2_ in Figure 4 (left panel) show clear differences before and after the annealing: the V2p _3/2_ peak of the annealed sample b_2_ shifts to a higher oxidation state and becomes sharper compared to the as-grown sample b_1_ due to the oxidization and crystallization upon post-annealing. O1s-V2p spectra and curve-fitting results of the samples a_1_ and a_2_ are presented separately in Supplementary Figure S1 and Table S1. Similar to sample b_2_, its V2p _3/2_ peak shows a clear shift to a higher oxidation state after annealing. Compared to sample a_1_, the V2p^2+/1+^_3/2_ peak disappears and the amount of V^5+^ increases significantly in sample a_2_. O1s-V2p spectra and curve-fitting results of the annealed films b_2_-d_2_ are presented in Figure S3 (left). All annealed films exhibit the same spectrum shape. Curve fitting [31,36,37] analysis (Figure S3 (left) and Table S3) confirms that the annealed samples have a similar surface chemical composition with the main peak at 517.3 (±0.1) eV that can be associated with V^5+^, and a smaller shoulder peak at 515.7 (±0.2) eV that can be associated with V^4+^. These XPS results further support the interpretation of the STM topographies as V_2_O_5_ and V_6_O_13_ (see Figure 3d,g). Note that surface reduction during the XPS analysis could also contribute partially to the intensity of the shoulder [38]. However, we did not observe any significant change in the V2p-spectra in repeated XPS measurements. We can conclude that all films (a_2_–d_2_) obtain a similar V2p _3/2_ peak shape with a mixed oxidation state after the annealing: V^5+^ predominates with a smaller fraction of V^4+^, which implies that the annealed films possess a chemical composition closer to bulk V_2_O_5_ rather than bulk V_6_O_13_ (516.5 eV [29]). Finally, we note that the slightly higher intensity of the annealed sample d_2_ implies that it is thicker than sample c_2_, which may be related to a higher effective sticking coefficient upon deposition of V in the presence of O_2_.

Core-level C1s spectra of samples b_1_ and b_2_ are shown in Figure 4 (middle panel). Both have a similar shape with mainly a strong C-C peak located at 284.5 eV. The tiny bump appearing in sample b_2_ at the position around 283.8 eV could originate from charge transfer owing to the oxide layer on top of graphene [39]. Note that binding energies around 282.2 eV (C1s) and 513.2 eV (V2p _3/2_) of vanadium carbide [40] are not detected in our samples. Together with the core level O1s-V2p and C1s spectra in Figure 4 (left and middle), we can conclude that no clear oxidation of HOPG substrate or formation of vanadium carbide occurs in the as-grown and annealed samples.

The XPS valence band (VB) spectra of samples b_1_ and b_2_ are presented in Figure 4 (right panel). Both spectra exhibit a broadband situated at ~3–9 eV stemming from O2p. Interestingly, we find a V3d peak located at the Fermi level in the as-grown sample b_1_, whereas sample b_2_ possesses an empty V3d state [41], indicating that vanadium oxide becomes more insulating upon post-annealing. VB spectra of the other annealed films (c_2_ and d_2_) are added in Figure S3 (right). They show a similar shape comparable with that of a freshly cleaved V_2_O_5_(001) single crystal ([37] Figure 2) and the ultrathin VO*_x_* film on Au (111) ([34] Figure 5). From these spectra, the valence band maximum (VBM) can be estimated by the intersection of two extrapolated lines (fitted baseline and fitted line of linear portion of lower binding energy side). The derived value is about 2.6 eV for all films (b_2_, c_2_, and d_2_), which is slightly higher than that of bulk V_2_O_5_ (~2.3 eV). This indicates that the structure of the ultrathin films may not precisely match that of the bulk V_2_O_5_, although similar spectral features indicate they share common structural elements, for instance, the observed V_2_O_5_ (001)-type surface structure from STM. It is worth mentioning that the V3d of bulk V_6_O_13_ (bandgap near 0 eV) is not expected to be empty [42], which is different from the empty V3d concluded in Figure 4 (right). A possible reason may be that the base layer (V_6_O_13_-like structure) in these annealed films does not strictly match the structure and property of bulk V_6_O_13_ owing to its very thin character. Theoretical calculations are obviously required to obtain an in-depth understanding of the electronic structure of the film, which is the scope of our future research. Nevertheless, our experiments demonstrate that a 2D insulating vanadium oxide can be grown directly on HOPG, suggesting that vanadium oxide has great potential for graphene/oxide heterostructures.

## 4. Conclusions

The key finding of this work is the formation of a 2D vanadium oxide monolayer on HOPG by depositing metal V and the subsequent post-annealing. The growth of V and VO*_x_* on HOPG has been systematically studied. Although V shows 3D growth on the HOPG surface, we find that oxygen can promote the formation of 2D VO*_x_* species possibly because it suppresses the clustering of V atoms. Depending on the coverage of vanadium oxide film, various morphologies are observed. Annealed sample at low coverage of VO*_x_* shows the morphology of 2D islands with small clusters on top, and those 2D islands preferentially accumulate near the step-edge of HOPG. By increasing the coverage of VO*_x_* film, a 2D vanadium oxide base layer is completed on HOPG. In thicker VO*_x_* films, 3D island growth starts on top of the monolayer. It is found that the monolayer has a V_6_O_13_(001)-like structure and the islands that appear in thick films possess a V_2_O_5_(001)-type structure. Note that those structures do not strictly correspond to any known bulk structures due to the relaxation or reconstruction of the ultrathin oxide film on the HOPG substrate. The chemical composition of the annealed films is studied by XPS and shows a mixed oxidation state of V^5+^ and V^4+^ which is in good agreement with STM observations. Moreover, the vanadium oxide is insulating, and the VBM is calculated to be around 2.6 eV which makes it a promising candidate for graphene/oxide heterostructure electronic devices in future applications. Applying the optimized growth recipe to single/multi-layer graphene surfaces, such as CVD-grown graphene on Cu, or graphene transferred to silicon substrates for transport measurements and FET devices will be the scope of our future research. Note that the rough surface of the CVD-grown graphene and the presence of polymer and metal catalyst residues make it challenging for achieving that goal.

## Figures and Tables

**Table 1:** Deposition parameters of the as-grown V or VO*_x_* films prepared under different conditions. V-thickness is calculated from the deposition rate (0.001 nm/s; calibrated via a QCM) and the deposition time.

Sample	a_1_	b_1_	c_1_	d_1_
V-thickness (nm)	0.03	0.3	0.45	0.45
O_2_ partial pressure	No O_2_	No O_2_	No O_2_	1 × 10^−6^ mbar

## Data Availability

The data presented in this study are available from the corresponding author upon request.

## Supplementary Materials

The following supporting information can be downloaded at: https://www.mdpi.com/article/10.3390/nano12183134/s1, Figure S1: XPS core-level V2p and O1s spectra and curve-fitting results of (top) as-grown sample a_1_ and (bottom) annealed sample a_2_. The fitting parameters are listed in Table S1. The minor discrepancy near 520 eV between the experimental values and the fitting result may be related to the existence of additional satellite peaks that are not included in the fitting model [31,36,37]; Figure S2: Plane-view TEM images for as-grown sample c_1_, (left) large-scale bright-field TEM image, the dark/grey spots confirm the island morphology; and (right) high-resolution TEM close-up view, indicating its mainly with an amorphous structure; Figure S3: (left) XPS core-level O1s-V2p spectra and curve-fitting results of the annealed films b_2_–d_2_. Note that ‘sat’ refers to so-called satellite peaks, as discussed in detail in Ref. [37]. The fitting parameters are listed in Table S3. (right) XPS valence band spectra and curve-fitting results of the annealed films b_2_–d_2_; Table S1: XPS fit parameters for the V2p and O1s signals of samples a_1_ and a_2_ (before and after annealing); Table S2: RMS roughness of the as-grown and the Ar-annealed samples. The value of each sample is calculated by averaging the RMS roughness of three AFM images (size 1 × 1 µm^2^) of the sample, and the indicated error is the corresponding standard deviation; Table S3: XPS fit parameters for the V2p and O1s signals of annealed sample b_2_–d_2_.
