# Supplementary material for: Growth and Characterization of Ultrathin Vanadium Oxide Films on HOPG
Source: Nanomaterials (Basel). 2022 Sep 9;12(18):3134. doi: 10.3390/nano12183134 (PMC9505911; doi:10.3390/nano12183134)
Supplement: Supplementary file 1 [file nanomaterials-12-03134-s001.zip › nanomaterials-1867653-supplementary.pdf]

nanomaterials

Supplementary Materials

Growth and Characterization of Ultrathin Vanadium Oxide Films on HOPG

Yue Sun [1], Koen Schouteden [2], María Recaman Payo [2], Jean-Pierre Locquet [2] and Jin Won Seo [1,*]

[1] Department of Materials Engineering, KU Leuven, Kasteelpark Arenberg 44, B-3001 Leuven, Belgium
[2] Department of Physics and Astronomy, KU Leuven, Celestijnenlaan 200D, B-3001 Leuven, Belgium
* Correspondence: maria.seo@kuleuven.be

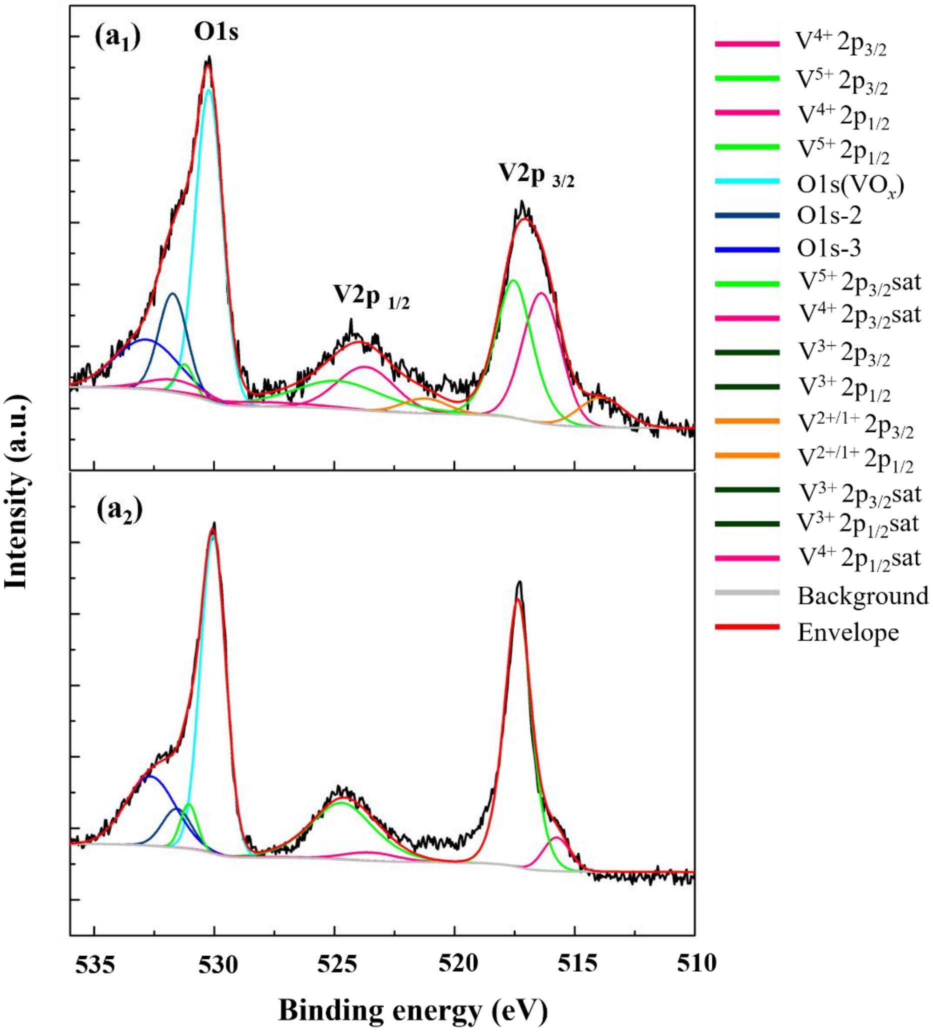

Figure S1. XPS core-level V2p and O1s spectra and curve-fitting results of (**top**) as-grown sample a_1 and (**bottom**) annealed sample a_2. The fitting parameters are listed in Table S1. The minor discrepancy near 520 eV between the experimental values and the fitting result may be related to the existence of additional satellite peaks that are not included in the fitting model [31,36,37].

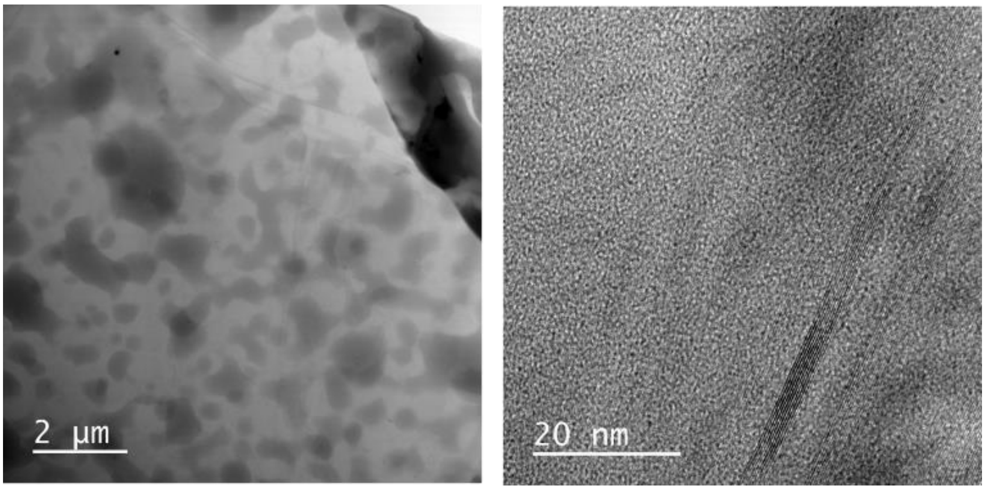

Figure S2. Plane-view TEM images of as-grown sample c_1, (**left**) large-scale bright-field TEM image, the dark/grey spots confirm the island morphology; and (**right**) high-resolution TEM close-up view, indicating its mainly with an amorphous structure.

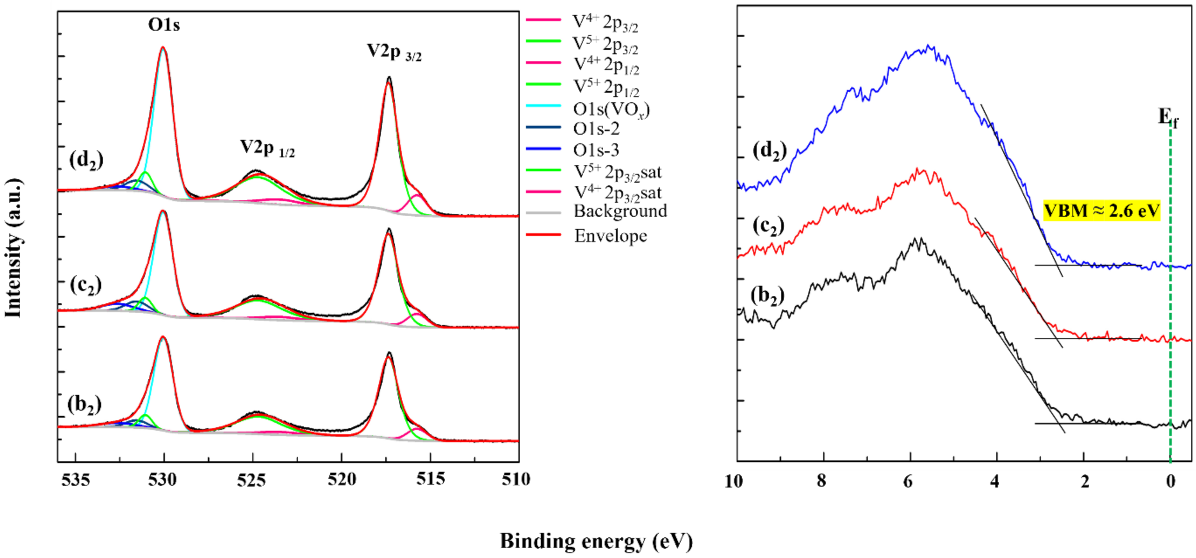

Figure S3. (**left**) XPS core-level O1s-V2p spectra and curve-fitting results of the annealed films b_2 - d_2. Note that 'sat' refers to so-called satellite peaks, as discussed in detail in Ref. [37]. The fitting parameters are listed in Table S3. (**right**) XPS valence band spectra and curve-fitting results of the annealed films b_2 -d_2.

Table S1. XPS fit parameters for the V2p and O1s signals of samples a_1 and a_2 (before and after annealing).

Core line	BE (eV)	FWHM (eV)		%Area	
		a_1	a_2	a_1	a_2
$V^{4+}2p_{3/2}$	515.7 (±0.2)	1.94	1.34	9.23	1.90
$V^{5+}2p_{3/2}$	517.3 (±0.1)	1.54	1.34	6.74	13.99
$V^{4+}2p_{1/2}$	523.5 (±0.2)	2.87	2.68	4.64	0.95
$V^{5+}2p_{1/2}$	524.6 (±0.1)	4.81	3.24	3.39	7.03
O1s (V-O)	530.0 (±0.1)	1.35	1.24	46.82	48.07
O1s-2	531.5 (±0.2)	1.25	1.44	11.72	6.88
O1s-3	532.6 (±0.2)	1.92	2.35	9.65	19.57
$V^{5+}2p_{3/2}$Sat.	531.0 (±0.2)	0.70	0.85	0.68	1.41
$V^{4+}2p_{3/2}$Sat.	528.0 (±0.2)	5.50	5.50	0.93	0.19

$V^{3+}2p_{3/2}$	515.3 (±0.2)	5.00	0.10
$V^{3+}2p_{1/2}$	522.6 (±0.2)	5.00	0.05
$V^{2+/1+}2p_{3/2}$	513.7 (±0.2)	1.53	1.21
$V^{2+/1+}2p_{1/2}$	521.0 (±0.2)	2.11	0.61
$V^{3+}2p_{3/2}$Sat.	526.4 (±0.2)	5.50	0.01
$V^{3+}2p_{1/2}$Sat.	531.3 (±0.2)	0.20	0.01
$V^{4+}2p_{1/2}$Sat.	531.4 (±0.2)	1.41	1.40

BE are referenced to the O1s (V-O) signal taken at 530.0 eV [29].
Sat. refers to satellite peaks [37].

Table S2. RMS roughness of the as-grown and the Ar-annealed samples. The value of each sample is calculated by averaging the RMS roughness of three AFM images (size 1 × 1 μm^2) of the sample, and the indicated error is the corresponding standard deviation.

as-grown	a_1	b_1	c_1	d_1
RMS (nm)	0.38 (± 0.1)	0.42 (± 0.2)	0.83 (± 0.1)	0.15 (± 0.1)
annealed	a_2	b_2	c_2	d_2
RMS (nm)	0.23 (± 0.1)	0.35 (± 0.1)	0.44 (± 0.1)	0.86 (± 0.1)

Table S3. XPS fit parameters for the V2p and O1s signals of annealed sample b_2-d_2.

**Core line**	**BE (eV)**	**FWHM (eV)**			**%Area**		
		**b_2**	**c_2**	**d_2**	**b_2**	**c_2**	**d_2**
$V^{4+}2p_{3/2}$	515.7 (±0.2)	1.30	1.30	1.26	2.28	2.33	2.50
$V^{5+}2p_{3/2}$	517.3 (±0.1)	1.30	1.30	1.26	17.03	16.55	16.75
$V^{4+}2p_{1/2}$	523.5 (±0.2)	2.60	2.61	2.51	1.15	1.17	1.26
$V^{5+}2p_{1/2}$	524.6 (±0.1)	2.99	3.14	3.09	8.56	8.32	8.42
O1s (V-O)	530.0 (±0.1)	1.26	1.25	1.25	59.30	56.84	60.84
O1s-2	531.5 (±0.2)	1.51	1.58	1.58	5.84	7.13	5.29
O1s-3	532.6 (±0.2)	2.24	2.03	2.30	3.89	5.75	2.99
$V^{5+}2p_{3/2}$Sat.	531.0 (±0.2)	0.86	0.86	0.88	1.72	1.67	1.69
$V^{4+}2p_{3/2}$Sat.	528.0 (±0.2)	5.50	5.50	5.50	0.23	0.23	0.25

BE are referenced to the O1s (V-O) signal taken at 530.0 eV [29] Sat. refers to satellite peaks [37].
